# Intracellular Serotonin Modulates Insulin Secretion from Pancreatic β-Cells by Protein Serotonylation

**DOI:** 10.1371/journal.pbio.1000229

**Published:** 2009-10-27

**Authors:** Nils Paulmann, Maik Grohmann, Jörg-Peter Voigt, Bettina Bert, Jakob Vowinckel, Michael Bader, Maša Skelin, Marko Jevšek, Heidrun Fink, Marjan Rupnik, Diego J. Walther

**Affiliations:** 1 Department of Human Molecular Genetics, Max Planck Institute for Molecular Genetics, Berlin, Germany; 2 Department of Biology, Chemistry, and Pharmacy, Free University Berlin, Berlin, Germany; 3 Institute of Pharmacology and Toxicology of the School of Veterinary Medicine, Free University Berlin, Berlin, Germany; 4 Laboratory of Molecular Biology of Peptide Hormones, Max Delbrück Center for Molecular Medicine, Berlin, Germany; 5 Institute of Physiology of the Medical Faculty, University of Maribor, Maribor, Slovenia; University of Cambridge, United Kingdom

## Abstract

While serotonin (5-HT) co-localization with insulin in granules of pancreatic β-cells was demonstrated more than three decades ago, its physiological role in the etiology of diabetes is still unclear. We combined biochemical and electrophysiological analyses of mice selectively deficient in peripheral tryptophan hydroxylase (*Tph1*−/−) and 5-HT to show that intracellular 5-HT regulates insulin secretion. We found that these mice are diabetic and have an impaired insulin secretion due to the lack of 5-HT in the pancreas. The pharmacological restoration of peripheral 5-HT levels rescued the impaired insulin secretion in vivo. These findings were further evidenced by patch clamp experiments with isolated *Tph1*−/− β-cells, which clearly showed that the secretory defect is downstream of Ca^2+^-signaling and can be rescued by direct intracellular application of 5-HT via the clamp pipette. In elucidating the underlying mechanism further, we demonstrate the covalent coupling of 5-HT by transglutaminases during insulin exocytosis to two key players in insulin secretion, the small GTPases Rab3a and Rab27a. This renders them constitutively active in a receptor-independent signaling mechanism we have recently termed serotonylation. Concordantly, an inhibition of such activating serotonylation in β-cells abates insulin secretion. We also observed inactivation of serotonylated Rab3a by enhanced proteasomal degradation, which is in line with the inactivation of other serotonylated GTPases. Our results demonstrate that 5-HT regulates insulin secretion by serotonylation of GTPases within pancreatic β-cells and suggest that intracellular 5-HT functions in various microenvironments via this mechanism in concert with the known receptor-mediated signaling.

## Abstract 2

Non-neuronal, peripheral serotonin deficiency causes diabetes mellitus and identifies an intracellular role for serotonin in the regulation of insulin secretion.

## Author Summary

Diabetes is the most prevalent metabolic disease and one that affects individuals of every social and economic status. The disease can arise as a result of reduced secretion of insulin from pancreatic β-cells or reduced action of insulin on its target organs. Therefore, understanding how to prevent and treat diabetes requires an extensive knowledge of the regulation of insulin secretion. In this study, we identify the hormone serotonin as a new regulator of insulin secretion and thereby attribute a function to the co-localization of serotonin and insulin in pancreatic β-cells that was first observed 30 years ago but until now not understood. We first demonstrate that a lack of serotonin in β-cells of transgenic mice leads to reduced insulin secretion and diabetes mellitus and that pharmacological replenishment of serotonin rescues insulin secretion in these mice. Interestingly, serotonin mainly acts not as an intercellular signaling molecule via its traditional surface receptors but intracellularly via regulation of the activity of target proteins through covalent coupling of serotonin to them. This coupling, called serotonylation, activates specific small GTPases, which in turn promote glucose-mediated insulin secretion. Adding this receptor-independent signaling mechanism to the multifarious regulatory functions of serotonin, we hypothesize that protein serotonylation modulates physiological secretion processes in all serotonin-containing tissues.

## Introduction

Diabetes mellitus, primarily defined as a chronic hyperglycemia giving rise to risk of microvascular damage, is one of the most serious metabolic disorders by means of 171 million people affected worldwide in 2000 and a projected 366 million by 2030 [1]. A comprehensive understanding of the molecular processes underlying the development of diabetes is of clinical and economic importance.

Several animal models for type 1 and 2 diabetes mellitus (T1DM, T2DM) and maturity-onset diabetes of the young (MODY) have been generated in order to shed light on the etiology of this disease [2]–[4], but many regulatory aspects of insulin secretion are poorly understood, as the machinery involved is complex. One issue that has eluded researchers for more than three decades concerns the role of serotonin (5-hydroxytryptamine; 5-HT) in β-cells [5],[6]. 5-HT is synthesized within β-cells [7], it is stored together with insulin in their secretory β-granules [8], and it is co-released when pancreatic islets are stimulated with glucose [9],[10]. For these reasons, insulin secretion is currently often monitored in cell models, such as insulinoma cells or freshly isolated islets, using 5-HT as a surrogate measure because of its rapid and reliable detection by electrophysiological techniques [7],[9],[10]. Nevertheless, the physiological meaning of 5-HT co-localization with insulin in β-cells remains a mystery.

A key to the understanding are recent advances in our knowledge of intracellular 5-HT functions, for instance during mammary gland involution, primary hemostasis, or liver regeneration [11]–[13], which have led to the concept of “microserotonergic systems” in peripheral tissues in contrast to the traditional view of 5-HT as a pleiotropic hormone. Studies on tryptophan hydroxylase 1 knockout mice (*Tph1*−/−), which are deficient in peripheral 5-HT but have normal 5-HT levels in the brain, identified the “serotonylation” of small GTPases as a receptor-independent, intracellular signaling mechanism of monoamines [13]. In thrombocytes, intracellular Ca^2+^ mobilization and monoamine accumulation in the cytoplasm trigger vesicular exocytosis through a constitutively activating covalent binding of 5-HT to GTPases of the Rho and Rab families, in a reaction that is conferred by the Ca^2+^-dependent transglutaminases (TGases) [13]. This process has been found also in smooth muscle cells, where the serotonylation of RhoA triggers their mitogenesis [14], and in platelets, where the serotonylation of Rab4 triggers the internalization of the 5-HT reuptake transporter (SERT) [15].

In addition, it has been suggested that TGase2 is involved in the glucose-stimulated insulin release from β-cells, based on the finding that *TGase2*−/− mice are glucose intolerant [16]. Furthermore, GTPases are crucial regulatory components in insulin secretion [17]–[19]. This prompted us to investigate serotonylation of GTPases as a modulatory mechanism in insulin secretion.

## Results/Discussion

### *Tph1*−/− Mice Lack 5-HT in the Pancreas and Are Diabetic

**Figure 1:**
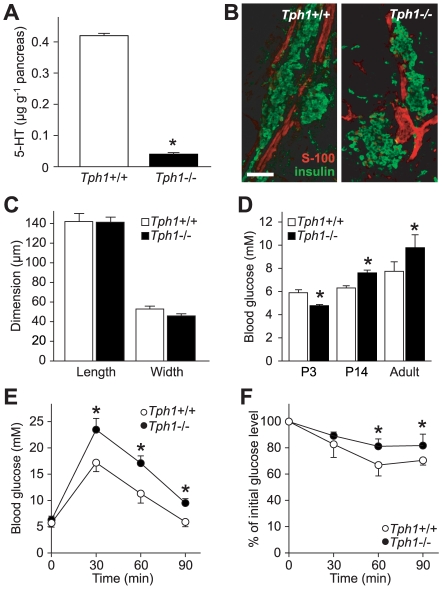
Pancreata of *Tph1*−/− mice have a normal morphology but lack 5-HT, which results in hyperglycemia and insulin resistance. (A) 5-HT contents in *Tph1*−/− and wt mice show, that *Tph1*−/− pancreata contain only around 10% of the wt mean. *p<0.05; *n* = 6. (B) Combined stacks of confocal images of pancreas slices spanning 10 µm with anti-insulin-stained islets (green) and anti-S100-stained ductal structures (red) at postnatal day 3 (P3) reveal no difference between the genotypes; scale bar = 70 µm. (C) Individual islets do not differ in morphometric analysis (*Tph1*−/−: *n* = 29; wt: *n* = 30). (D) Basal blood glucose concentration in freely feeding *Tph1*−/− mice are significantly reduced at P3 (*Tph1*−/−: *n* = 20; wt: *n* = 11) and elevated at P14 (*n* = 5) and in adulthood (*n* = 6) compared to wt littermates. *p<0.05. (E) Glucose tolerance tests with fasted wt and *Tph1*−/− mice show impaired glucose tolerance of the latter. *p<0.05; *n* = 6. (F) Insulin tolerance tests with *Tph1*−/− and wt mice. *p<0.05; *n* = 6. For (A) and (C–F), data are means ± SEM.

Pancreatic 5-HT levels in *Tph1*−/− mice only reach 10% of the wild-type (wt) mean (Figure 1A) and neither pancreas mass (*Tph1*+/+: 150±30 mg, *n* = 27; *Tph1*−/−: 160±40 mg, *n* = 30) nor morphology, nor the number and size of islets differ between *Tph1*−/− and wt mice (Figure 1B and 1C). Concordant to the lack of peripheral 5-HT in *Tph1*−/− mice, they are hypersensitive to the satiety mediating effect of systemically applied 5-HT (Figure S1). In addition, the basal blood glucose concentrations in freely feeding mice are significantly elevated relative to those in wt mice (Figure 1D), while none of the other pancreatic markers in extensive serological screenings are altered in *Tph1*−/− mice (Table S1). The glucose levels are elevated as early as 14 d after birth in *Tph1*−/− mice and are maintained into young adulthood, indicating glucose intolerance. Concordantly, fasted *Tph1*−/− mice exhibited significantly higher blood glucose concentrations than wt animals in glucose tolerance tests (Figure 1E). Young adult *Tph1*−/− mice also present a mild insulin resistance (Figure 1F).

**Figure 2:**
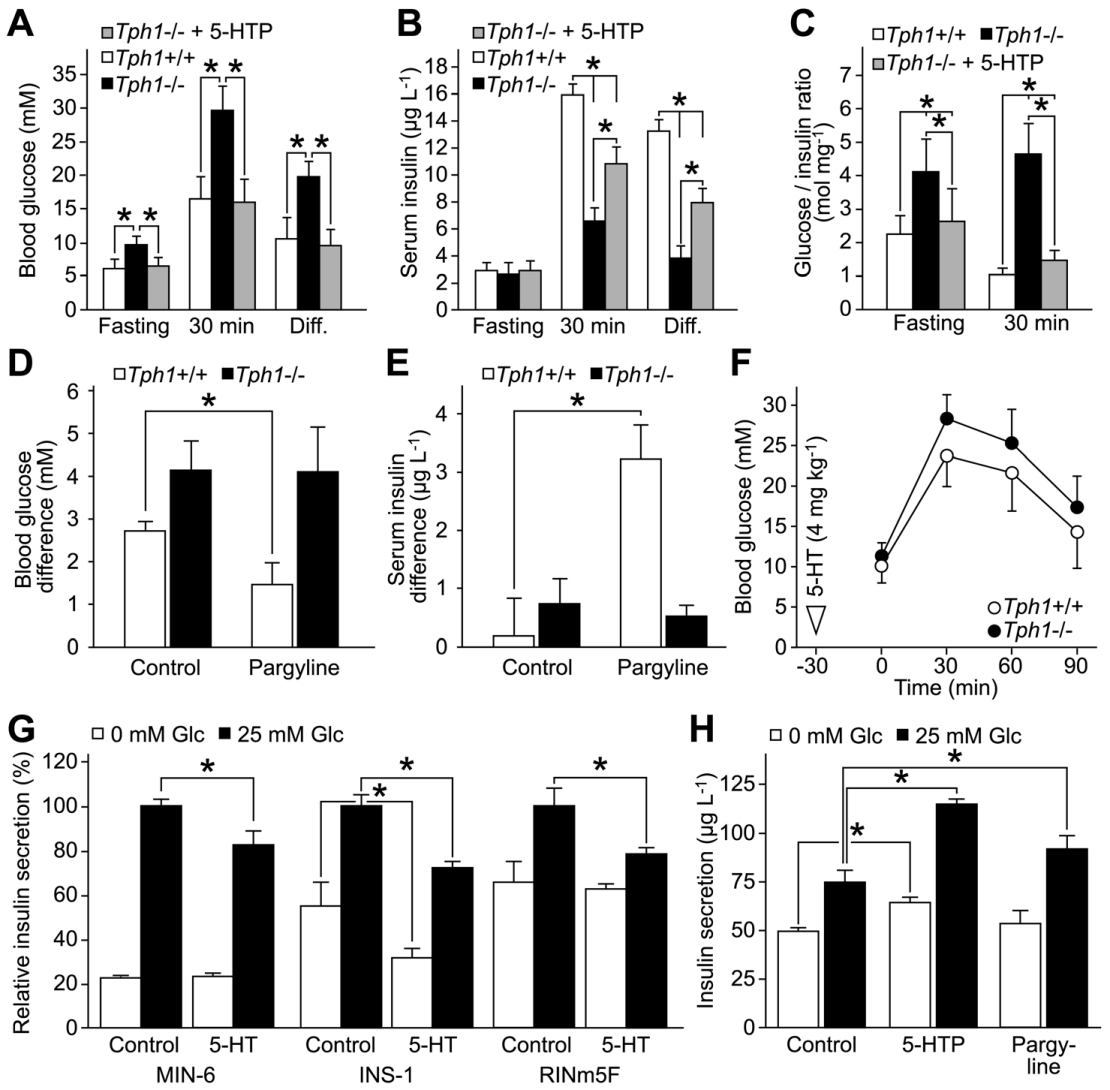
Intra- and extracellular 5-HT modulates insulin secretion in opposing directions. (A–C) Diabetes mellitus and β-cell dysfunction in *Tph1*−/− mice aged 64 to 70 wk and rescue with 5-HTP. Glucose tolerance test with simultaneous blood glucose (A) and serum insulin (B) measurements. Glucose to insulin ratios (C) are significantly increased in *Tph1*−/− without 5-HTP treatment before and after the glucose load. *p<0.05; *n* = 8 (wt and *Tph1*−/− + 5-HTP) and *n* = 6 (*Tph1*−/−). (D) Pargyline induces hyperglycemia in wt mice. After a meal of 60 min, mice were treated with pargyline (75 mg kg^−1^) and blood glucose was measured immediately and after additional 60 min. *p<0.05; *n* = 6. (E) Pargyline treatment substantially elevated insulin secretion in wt mice. Mice were treated as described in (D). *p<0.05; *n* = 6. (F) Glucose tolerance test of *Tph1*−/− and wt mice with systemic 5-HT pre-treatment. *p<0.05; *n* = 6. (G) Extracellular 5-HT inhibits insulin secretion in MIN-6, INS-1, and RINm5F insulinoma cells. Cells were treated with 5-HT (500 µM) at the beginning of a 60 min secretion period. Insulin secretion is normalized to glucose-induced control cells. *p<0.05; *n* = 4. (H) Insulin secretion of RINm5F cells with or without glucose and 5-HTP (500 µM; 3 h) or pargyline (20 µM; 3 h). *p<0.05; *n* = 3. All data are presented as means ± SEM.

The glucose intolerance is comparable to the T2DM phenotype of 5-HT_2C_ receptor knockout mice [20] and occurs at a surprising young age between postnatal Days 3 and 14 (Figure 1D). Glucose tolerance tests with simultaneous glucose and insulin measurements then revealed a primary β-cell dysfunction in glucose-induced insulin secretion (Figure 2). The rise in blood glucose was significantly higher in the *Tph1*−/− mice 30 min after a glucose load (Figure 2A) and was not proportional to the initially elevated glucose level (difference significantly higher). The glucose load was also accompanied with a significantly shortened insulin secretion (Figure 2B), best seen at the difference. Therefore, glucose to insulin ratios (Figure 2C) were significantly increased in *Tph1*−/− before and after the glucose load, which indicates a β-cell dysfunction. This reaction was also observed in younger animals of both mixed and inbred genetic backgrounds (Figure S2). Moreover, the fasting glucose levels were increased by around 20% to 9.7 mM in the *Tph1*−/− aged 64–70 wk (Figure 2A). In conclusion, *Tph1*−/− mice are clearly diabetic, but the classification of the specific subtype of diabetes is difficult due to the lack of standardized mice-specific criteria. Nevertheless, such a phenotype, a primary β-cell dysfunction in glucose-induced insulin secretion with a monogenic inheritance and an early onset but with no loss of β-cell mass, would be classified as MODY in humans [21]. Therefore, one might speculate that *TPH1* is a possible candidate gene for this metabolic disorder in humans, especially as a genetic diagnosis in at least 11% of European and as many as 70%–80% of Asian families with a diagnosis of MODY cannot be made, indicating that additional MODY-related genes need to be identified [22]–[24]. Nonetheless, follow-up screening of the *TPH1* gene of unclassified MODY patients is to be performed in order to support this hypothesis.

Interestingly, *Tph1*−/− mice thrive normally [13],[25], and they live as long as their wt littermates (mean life expectancy: 113 wk; *n* = 37; χ^2^ = 0.98). We consider these findings to be particularly important, since diabetes mellitus, if left untreated, significantly curtails life expectancy [21],[26]. Especially the development of secondary damage like cardiovascular disease is the major cause for morbidity and mortality in diabetes. We have previously demonstrated that *Tph1*−/− mice are less prone to succumb to vessel occlusion in experimental thromboembolism and thrombosis due to a reduced primary haemostatic response [13]. This beneficial protective side effect of the lack of 5-HT decreases cardiovascular complications in *Tph1*−/− mice and seems to mild the severity of their diabetes. The general differences between mouse and man hamper a direct translation of findings between these species. Nevertheless, one might speculate that therapeutic peripheral 5-HT reduction specifically in thrombocytes [13],[27], together with blood glucose management, can also ameliorate vascular disease and its complications in diabetic patients. This concept for future work could possibly open new avenues to improve quality of life and to prolong life expectancy of diabetic patients.

### Extra- and Intracellular 5-HT Modulates Insulin Secretion in Opposing Directions

To further elucidate the role of 5-HT in diabetes, we took advantage of well-established pharmacological possibilities for manipulating intracellular 5-HT levels (Figure S3). Application of 5-hydroxytryptophan (5-HTP), the immediate precursor of 5-HT (Figure S3A), increases the intracellular 5-HT level, bypassing the rate-limiting biosynthesis step [6],[11],[28]. Indeed, this treatment normalized the blood glucose levels and largely rescued the deficient insulin secretion in the *Tph1*−/− mice (Figure 2A and 2B).

In addition to the use of 5-HTP to increase intracellular 5-HT, pargyline, which blocks the 5-HT catabolism [6], can also be used for this purpose (Figure S3A). Systemic application of pargyline leads to a rapid hypoglycemic and hyperinsulinemic response in mice [29], suggesting that intracellular 5-HT enhances insulin secretion. If intracellular 5-HT plays such an enhancing role in insulin secretion, then pargyline application should have no effects in *Tph1*−/− mice, which lack 5-HT. Concordantly, pargyline induced hypoglycemia (Figure 2D) and an acute rise in plasma insulin (Figure 2E) exclusively in wt but not in *Tph1*−/− mice.

We then investigated glucose homeostasis after a systemic 5-HT application 30 min prior to the glucose load in fasted mice, in order to explore the extracellular role of 5-HT. In these tests, mice of both genotypes had largely elevated blood glucose levels (Figure 2F) compared to mice without pre-treatment (Figure 1E). This suggests that extracellular 5-HT has an inhibitory influence on insulin secretion as noted previously [30], most likely acting via the sole known 5-HT receptor of β-cells, the 5-HT_1A_ receptor.

The effect of extracellular 5-HT on insulin secretion of β-cells was further investigated using the insulin-secreting cell lines MIN-6, INS-1, and RINm5F [31],[32], which all exhibit responsiveness to glucose-stimulation (Figure 2G). Indeed, extracellular 5-HT inhibited glucose-induced insulin secretion in all three β-cell lines. One has to take into account that β-cells take up 5-HT via the plasma membrane SERT into the cytoplasm [7],[9],[10]. Therefore, 5-HT might also partially act intracellularly in this experiment. But since the incubation with 5-HT was short, its action can be attributed mostly to extracellular mechanisms.

Moreover, elevated levels of intracellular 5-HT would rather not inhibit but enhance the glucose-mediated insulin secretion, as seen by 5-HTP and pargyline treatment of insulinoma cells (Figure 2H). The stronger effect of 5-HTP found in these tests is supported by our finding that 5-HTP rises the intracellular 5-HT concentration to a higher extent than pargyline treatment (Figure S4). High intracellular 5-HT levels seem to be involved in the control of basal secretion as well, as 5-HTP slightly enhances insulin secretion from RINm5F cells also under non-stimulatory conditions (Figure 2H). The hyperinsulinemic effect of the above-mentioned compounds that increase intracellular 5-HT levels is in accordance with the results in vivo (Figure 2B and 2E), further supporting a crucial role for intracellular 5-HT as a modifier of insulin secretion.

### Impaired Secretory Activity from β-Cells in Adult *Tph1*−/− Mice

**Figure 3:**
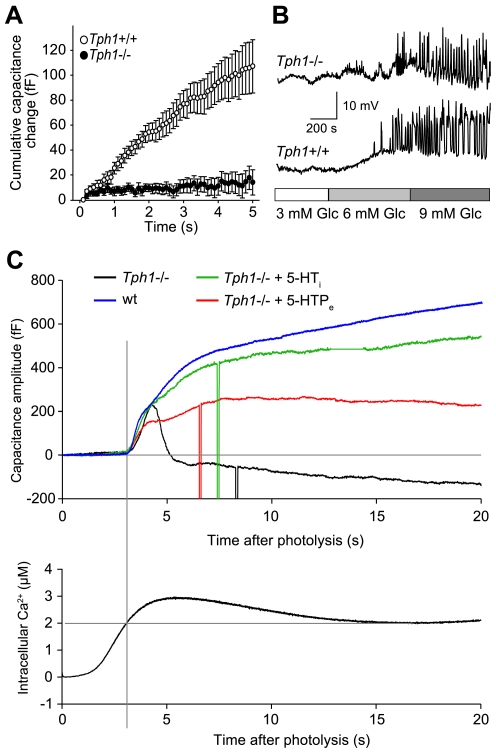
Impaired exocytosis of *Tph1*−/− β-cells and rescue with 5-HT. (A) A train of 50 depolarizing pulses from −80 to +10 mV for 40 ms at 10 Hz induced changes in the membrane capacitance of wt (*n* = 24) and *Tph1*−/− (*n* = 19) β-cells. Data are shown as means ± SEM. (B) Representative current-clamp recordings of the electrical activity of wt and *Tph1*−/− β-cells in pancreas tissue slices. The slices were perfused with solutions containing different glucose concentrations as indicated in the bar below the traces. (C) Representative membrane capacitance response of isolated wt and *Tph1*−/− β-cells (top panel) stimulated by ramp [Ca^2+^]_i_ change induced by slow photo-release of caged Ca^2+^ (bottom panel). The impaired component exocytosis has been partially rescued by extracellular 5-HTP (500 µM, 24 h) and completely restored by pipette intracellular dialysis with 5-HT.

To directly test the hyposecretory phenotype, we monitored secretion from *Tph1*−/− and wt β-cells by whole-cell patch-clamp measurements of membrane capacitance in fresh pancreas slices, taking advantage of the fast time resolution of electrophysiological techniques [33]. The resting membrane capacitance, electrical coupling between neighboring cells, and the amplitude of high voltage-activated Ca^2+^ channels (VACC) did not differ significantly between the *Tph1*−/− and wt β-cells (Figure S5). Nevertheless, the secretory response in *Tph1*−/− β-cells was significantly impaired when triggered by depolarization trains and measured as a cumulative increase in membrane capacitance (Figure 3A). Impaired secretion can also be due to impaired function of high VACC, but we found that peak amplitude of high voltage-activated (HVA) Ca^2+^ currents were not significantly different (Figure S5A). However the amplitude of low voltage-activated (LVA) Ca^2+^ currents was prominently increased in *Tph1*−/− compared to wt β-cells (Figure S5B). The β-cell electrical activity in *Tph1*−/− cells seems to be activating at lower glucose concentration (Figure 3B), which likely results from the increased LVA Ca^2+^ current amplitudes we observe in these mice (Figure S5B), because increased T-type currents are known to lower the threshold for action potential firing in insulinoma cells (INS-1) and non-obese diabetic mice [34],[35]. As the HVA current amplitudes were not significantly impaired, we conclude that the secretory impairment is not due to lower Ca^2+^ influx. Next we tested the impairment of the secretory response downstream of the Ca^2+^ influx. Slow UV photo-release of caged Ca^2+^ increased intracellular Ca^2+^, which peaked after about 5 s and reached a steady state value after 10 s (Figure 3C, lower panel). When free Ca^2+^ exceeded about 2 mM, a biphasic response in membrane capacitance was triggered. The amplitude of the fast component was comparable in both phenotypes, whereas the amplitude of the slow component was significantly reduced in *Tph1*−/− (Figure 3C, upper panel; for statistics see Figure S6). To test the direct role of 5-HT, we either incubated isolated β-cells with 5-HTP for 24 h or infused 5-HT through the patch pipette. While 5-HTP treatment partially restores the capacitance response, infusion of 5-HT completely rescued the reduced secretory phenotype (Figure 3C, upper panel). We conclude that the secretory lesion does not involve the coupling of the secretory machinery to either VACCs or Ca^2+^ sensors but interferes with the recruitment and exocytosis of the dense-core vesicles. The process is fast since the cell dialysis with 5-HT rescues the phenotype within a few minutes.

### Serotonylation Modulates Insulin Secretion

**Figure 4:**
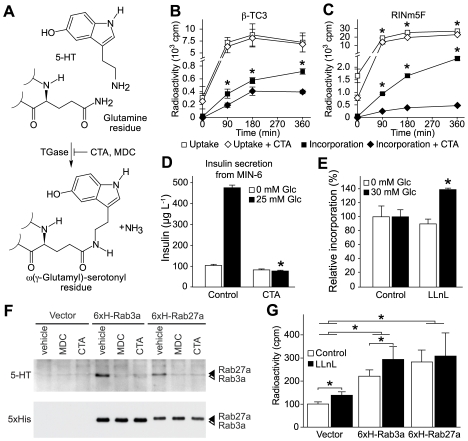
Inhibition of protein serotonylation reduces insulin secretion. (A) Scheme of protein serotonylation of glutamine residues by TGase. Cysteamine (CTA) and monodansylcadaverin (MDC) are potent inhibitors of TGases. (B and C) Uptake and protein incorporation of [^3^H]-5-HT in β-TC3 (B) and RINm5F cells (C) in the presence of CTA (500 µM; 3 h). *p<0.05; *n* = 3. (D) Insulin secretion from MIN-6 cells in the presence of CTA (500 µM; 3 h). *p<0.05; *n* = 4. (E) Proteasomal degradation of serotonylated proteins in RINm5F cells at different glucose concentrations in the presence or absence of the proteasomal inhibitor LLnL (50 µM; 3 h).*p<0.05; *n* = 4. (F) Immunoblot of serotonylated (5-HT) and total (5xHis) 6xH-Rab3a (white arrowhead) and 6xH-Rab27a (black arrowhead) prepared from glucose-stimulated RINm5F cells treated with 100 µM MDC, 200 µM CTA, or vehicle. Shown is one representative experiment out of three repetitions. (G) Quantification by Ni-NTA pull-downs of 6xH-Rab3a and 6xH-Rab27a from glucose-stimulated RINm5F cells incubated with [^3^H]-5-HT and either 50 µM LLnL or vehicle. *p<0.05; *n* = 3. Vector, vector-transfected control. For (B–E) and (G), data are means ± SEM.

In thrombocytes, intracellular 5-HT serves as a substrate for protein serotonylation (Figure 4A), which modifies the trafficking of proaggregatory α-granules and results in their exocytosis [13]. Thus, we asked whether serotonylation is also involved in the β-granule transport and exocytosis. We first confirmed that RINm5F and β-TC3 cells readily take up [^3^H]-5-HT (Figure 4B and 4C), as previously described for other insulinoma cell lines and islets [7],[9],[10]. The uptake has a similar kinetic in both cell lines, leveling off approximately 90 min after addition of [^3^H]-5-HT. Under these conditions, we detected covalent [^3^H]-5-HT incorporation into the protein fraction in both cell lines, revealing a massive serotonylation of proteins. This serotonylation was sensitive to the potent TGase inhibitor cysteamine in both cell lines, demonstrating that the reaction is TGase-dependent. More importantly, cysteamine also curtailed the glucose-stimulated insulin secretion in MIN-6 insulinoma cells (Figure 4D), as well as in INS-1 and RINm5F cells (Figure S7). These results are in line with the reduced insulin secretion of primary β-cells from rat treated with either the TGase inhibitor monodansylcadaverin (MDC) [36] or cysteamine [37] and the reduced insulin release and the MODY phenotype of *TGase2*−/− mice [16]. Thus, protein serotonylation is unequivocally detectable in β-cells and it modulates insulin secretion. Nonetheless, the further elucidation of signaling cascades regulating or interfering with this novel mechanism is an obvious issue that has to be clarified in follow-up studies.

Unexpectedly, the [^3^H]-5-HT incorporation rates in glucose-stimulated and non-stimulated RIN5mF cells did not differ (Figure 4E). However, it is now well-established that small GTPases constitutively activated by TGases are marked for proteasomal degradation by ubiquitination [14]. Concordantly, inhibition of the proteasome with calpain inhibitor I (LLnL) significantly accumulated serotonylated proteins only in the presence of glucose (Figure 4E), suggesting that with an increased insulin secretion, there is a faster cycling between activation by serotonylation and inactivation by proteasomal degradation.

### Rab3a and Rab27a Become Serotonylated during Glucose-Mediated Insulin Secretion

Previously, GTPases have been found to be targets for serotonylation in other tissues [13],[14], prompting us to test two GTPases crucially involved in insulin secretion as putative targets in β-cells; Rab3a regulates replenishment of the ready releasable pool of β-granules by recruiting calmodulin to the resting pool at early stages of vesicle transport [38]. Rab27a acts directly in the targeting of β-granules from the resting pool to the ready releasable pool at the plasma membrane [19]. To confirm their serotonylation during insulin secretion, we expressed 6xHis-tagged Rab3a and Rab27a in RINm5F cells (Figure S8), which express all components for serotonylation (Figure S9). Indeed, both GTPases become serotonylated within these cells in a TGase-dependent manner, as the TGase inhibitors MDC and cysteamine both blocked this reaction (Figure 4F). In addition, using radiolabeled [^3^H]-5-HT, we quantified the serotonylation of these GTPases (Figure 4G). LLnL enriched serotonylated Rab3a but not Rab27a, showing that proteasomal degradation is the mechanism of inactivation of Rab3a, at least at the selected time point, as shown for serotonylated RhoA [14]. The difference between the two GTPases could reflect different kinetics of inactivation by proteasomal degradation, suggesting a high selectivity of this process. While Rab3a-deficient mice present with glucose intolerance, transfection of insulinoma cells with constitutively active Rab3a was shown to inhibit insulin release by accumulating and retaining the vesicles in the ready releasable pool [17]. Hence, the continuous secretion seems to demand a rapid cycling of Rab3a between an active and inactive state. In contrast, constitutively active Rab27a has been shown to increase insulin release [39]. Thus, it could be of physiological relevance to serotonylate Rab27a to a higher extent and stability than Rab3a in order to promote continuous β-granule exocytosis.

**Figure 5:**
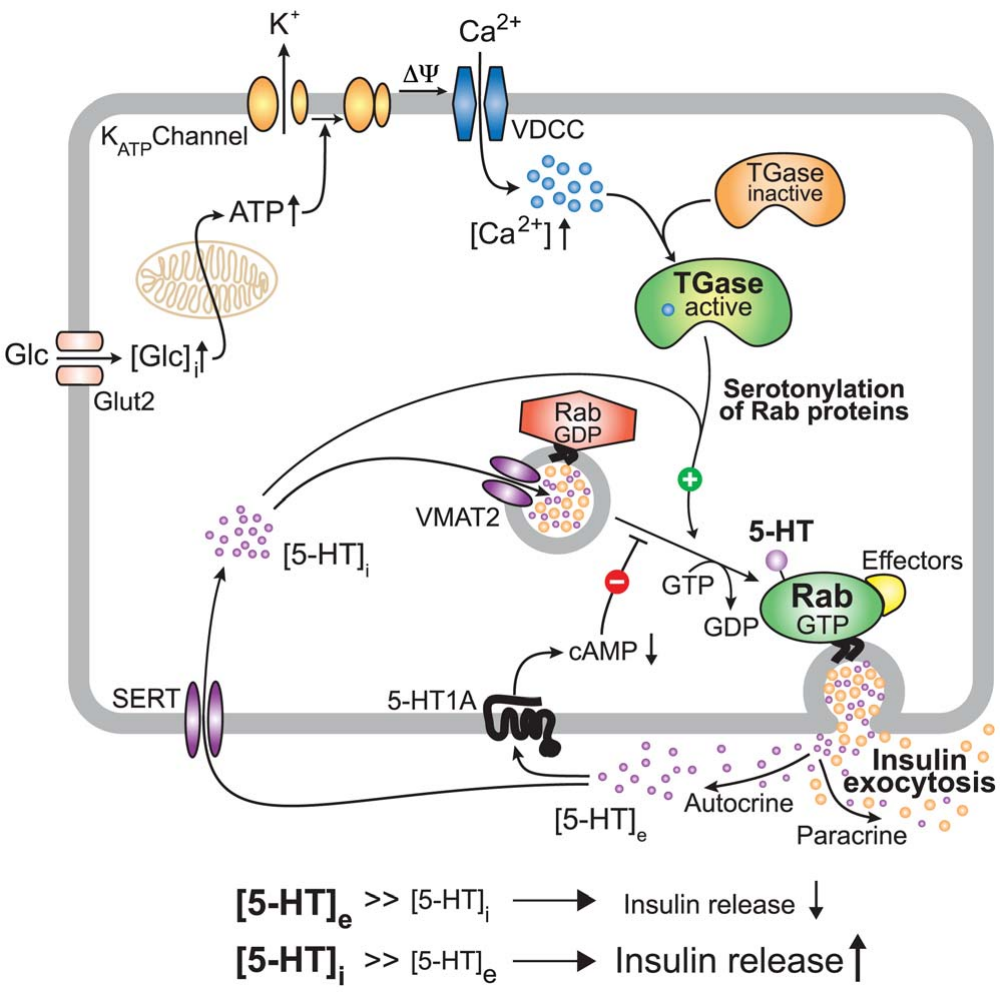
Proposed model of 5-HT-induced exocytosis of β-granules from glucose-stimulated β-cells. The classic sequel of glucose transport, glucose oxidation and ATP generation, ATP-gated potassium channel closure, membrane depolarisation, and Ca^2+^ influx via voltage-dependent calcium channels is represented in the upper part of the scheme. Amongst other functions, Ca^2+^ activates TGases that serotonylate a variety of proteins. In the β-cell, Rab3a, and Rab27a, which are crucially involved in the exocytosis of insulin, are target signaling proteins that become activated by this mechanism. Insulin and 5-HT co-secretion is the consequence. While insulin then exerts its endocrine function, 5-HT acts via an autocrine/paracrine loop. Initially, high extracellular 5-HT concentrations [5-HT]_e_ attenuate further insulin secretion through 5-HT_1A_ receptors, with progressive weakening of this effect by the clearance of [5-HT]_e_ via uptake through the plasma membrane SERT. Eventually, [5-HT]_i_ reaches much higher levels than [5-HT]_e_, promoting another event of insulin secretion by serotonylation. This 5-HT-dependent cycling between inhibition and induction might contribute to the well-known oscillating nature of insulin exocytosis from glucose-stimulated β-cells.

The data presented here suggest a model in which TGase is activated by the increase of intracellular Ca^2+^ that occurs upon glucose stimulation (Figure 5). TGases then use intracellular 5-HT to serotonylate Rab3a and Rab27a, likely amongst a set of diverse other yet to be identified target proteins, as suggested by the massive serotonylation observed in β-cells (Figure 4B and 4C). This renders them constitutively active and promotes insulin secretion. The serotonylated GTPases become inactivated by ubiquitin-dependent proteasomal degradation at different rates, which is analogous to the recently described inactivation of serotonylated RhoA [14].

This model is in line with the well-known pulsatile insulin secretion with a frequency of about one to five per minute, which is accompanied by synchronous intracellular Ca^2+^ oscillations [40]. These Ca^2+^ oscillations that are likely paralleled by intracellular 5-HT oscillations fulfill the conditions required for serotonylation of GTPases [13]. Furthermore, our data are in accordance with the phenotype of *TGase2*−/− mice, which also present a reduced β-cell function [16]. Thus, inactivation of the catalyzing enzyme TGase2 [16], a lack of the monoamine substrate for serotonylation (*Tph1*−/−), and the absence of the protein substrates Rab3a and Rab27a [18],[19] all lead to defective insulin secretion independently. These findings support an essential function of serotonylation for insulin exocytosis.

The data presented here assign a mechanistic role to the 5-HT contained in β-granules as a substrate for the serotonylation of proteins involved in insulin secretion. Our model, in which signaling protein serotonylation strengthens the function of the exocytotic machinery, shows striking resemblance to the process in thrombocytes [13]. Our findings suggest that serotonylation is a modifier mechanism in the high clinical variability of diabetes and may have an impact on future therapeutic concepts. If confirmed in other monoamine-rich cells, such as histaminergic or catecholaminergic neurons, this study has not only elucidated a key role of 5-HT in β-cells but also may contribute to change more general concepts about monoamine functions, adding another posttranslational modification to the repertoire of signaling.

## Materials and Methods

### Animal Experiments

Animals were held under standardized conditions on a mixed genetic 129/SvEvBrd × C57BL/6 background as previously described [13],[25]. In order to ensure that *Tph1*−/− and *Tph1*+/+ do not genetically drift away from each other, they were periodically interbred to obtain *Tph1*+/−, which were then used to re-establish *Tph1*−/− and *Tph1*+/+ to refresh the breeding colony, as generally recommended for mixed genetic backgrounds [41]. At the beginning of physiological experiments, the mice were at the F_11_-generation of interbreeding. In addition, *Tph1*−/− were backcrossed to the inbred strain C57BL/6 until the F_8_-generation and compared with the mixed genetic background. All phenotypical aspects were the same in the 129/SvEvBrd × C57BL/6 and the inbred C57BL/6 *Tph1*−/−. Pancreas extracts and whole blood samples for the determination of 5-HT contents were obtained and analyzed as described [25],[42]. For glucose and insulin measurements, blood of barbital sodium-anaesthetized animals was obtained from the *vena cava inferior*, the retrobulbar plexus, or by severing the tail tips. Glucose was immediately measured using a B-glucose analyzer (HemoCue) and insulin in serum or cell culture supernatants was quantified by insulin ELISA (Mercodia). Mice were fasted for 24 h prior to tolerance tests. Glucose (2 g kg^−1^), insulin (0.75 U kg^−1^), 5-HT (4 mg kg^−1^) or pargyline (75 mg kg^−1^) was injected intraperitoneally. Blood for serum preparation was collected from the *vena cava inferior* as described [13]. The serological analysis was performed at the animal clinic laboratory VetMedLab GmbH, Division of IDEXX Laboratories Inc. (Ludwigsburg, Germany). Peripheral 5-HT levels for rescue experiments were replenished by subcutaneous injections with 5-HTP as described [11].

### Pancreas Slices, Immunostaining, and Electrophysiology

The agarose embedded tissue (Seaplaque GTG agarose, BMA, Walkersville) was cut to 140 µm thick slices. During slicing and after the preparation, the tissue was kept in an ice-cold extracellular solution (125 mM NaCl, 2.5 mM KCl, 26 mM NaHCO_3_, 1.25 mM Na_2_HPO_4_, 2 mM sodiumpyruvate, 0.25 mM ascorbic acid, 3 mM myo-inositol, 6 mM lactic acid, 1 mM MgCl_2_, 2 mM CaCl_2_ and 3 mM glucose) adjusted to pH 7.3 by gassing with carbogen (95% O_2_, 5% CO_2_) for at least 30 min. The slices were transferred to carbogen-bubbled extracellular solution at 32°C 30 min before experiments. For primary β-cell culture, liberase (0.3 g L^−1^) (Roche) was dissolved in HBSS (Invitrogen) and injected into pancreas via the bile duct. The pancreas was removed and digested for 10–15 min at 37°C, centrifuged, and then hand picked [43]. Isolated islets were trypsinized into single cells, plated onto coverslips, and cultured in RPMI 1640 medium supplemented with 10% fetal bovine serum (Invitrogen) and antibiotics. The cultured β-cells were used within 3 d. For immunostaining, cells were incubated overnight at 4°C with anti-insulin and anti-S100 antibodies followed by anti-mouse Alexa 488 and anti-rabbit Alexa 647 for 1 h at room temperature. The fluorescence was acquired using a confocal microscope (Leica TCS SP2 AOBS) at 488 and 633 nm. The images were processed for morphometrical analysis using Leica confocal software. Pancreatic β-cells were identified by the characteristic half maximal inactivation of voltage-activated Na^+^ currents (approx. −100 mV) [44]. The pipette filling solution used in depolarization protocols, isolation of Ca^2+^ currents and current-clamp experiments on β-cells in slices contained 127 mM Cs-methanesulfonate, 8 mM CsCl, 10 mM Hepes (pH 7.2; CsOH), 2 mM MgCl_2_, 0.05 mM EGTA, 20 mM TEACl, and 4 mM ATP-Na_2_ (see [44] for details of the analysis). The pipette filling solution used in photo-release experiments on isolated β-cells contained 125 mM CsCl, 40 mM Hepes (pH 7.2; CsOH), 2 mM MgCl_2_, 0.05 mM EGTA, 20 mM TEACl, 2 mM ATP-Na_2_, 5 mM NP-EGTA (Molecular Probes), 4.5 mM CaCl_2_, and 0.1 mM Fura-6 (Molecular Probes). The perfusion chamber was mounted on an upright microscope (×60 water, NA 0.9, Eclipse E600FN, Nikon, Japan). All experiments were performed on a SWAM II C dual-phase lock-in patch-clamp amplifier (Celica, Ljubljana). Data were filtered at 3 kHz by an A/D converter (National Instruments). Recording, stimulation, and preliminary analysis were performed using the WinWcp software (v3.52, John Dempster, University of Strathclyde, UK). Patch pipettes were pulled (P-97; Sutter Instruments, USA) from borosilicate glass capillaries (GC150F-15; WPI, USA) to a resistance of 2–5 MΩ in pipette solution as reported previously. All currents were analyzed and presented after p/n leak subtraction. To estimate changes in membrane capacitance, the piecewise linear technique was used (1.6 kHz sine-wave frequency, 11 mV RMS amplitude). Secretory activity was triggered by elevations of cytosolic Ca^2+^. To manipulate Ca^2+^, we used depolarizing trains or continuous photo-release (Polychrome IV, Till Photonics) of caged Ca^2+^ (NP-EGTA). For data analysis and figure preparation, we used Matlab, Matview (Matlab WinWCP extension, Wise Technologies, Ljubljana), Sigmaplot, and Sigmastat (SPSS, Chicago, IL, USA).

### Cell Culture

Rat insulinoma cell line INS-1 was a generous gift from Claes B. Wollheim (University Medical Center, Geneva), and mouse MIN-6 cells were kindly provided by Franz Schaefer (University Heidelberg) and cultured as described [31],[32]. RINm5F cells were grown as previously described [45] in RPMI1640 (Invitrogen) containing 11.1 mM glucose supplemented with 10% fetal bovine serum (Biochrom) and antibiotics.

### Insulin Secretion Assays

Insulinoma cells were cultured in Primaria plates (Falcon) 72 h before the experiment and pretreated with 5-HTP, pargyline, or cysteamine as indicated in the figure legends. The cells were washed twice in glucose-free growth medium supplemented with dialyzed serum to remove 5-HT and sensitized to glucose for 30 min in glucose-free medium and stimuli. Next, cells were stimulated for 60 min with or without 25 mM glucose in KRBH (136 mM NaCl, 4.7 mM KCl, 1.2 mM MgSO_4_, 1 mM CaCl_2_, 1.2 mM KH_2_PO_4_, 5 mM NaHCO_3_, 10 mM HEPES, and 0.5% BSA, pH 7.4) in presence of indicated stimuli. We used KRBH to avoid any disturbances by compounds present in normal medium and/or serum (e.g., 5-HT). The supernatant was collected, centrifuged, and used for insulin ELISA as described above.

### Molecular Biological and Analytical Methods

All cloning procedures, PCR, and immunoblotting were conducted according to standard protocols or to the manufacturer's instructions. 6xH-Rab constructs were generated using Rab3a and Rab27a cDNAs [13] that were reamplified with 5′-primers containing an ATG within a Kozak consensus sequence followed by six histidine codons and a stretch of 22 specific nucleotides for each cDNA. Stable transfection of RINm5F cells was conducted with linearized constructs and the transfectants were selected at 50 µg mL^−1^ G418 for at least 4 wk.

### Quantitative Serotonylation Assay

TGase-mediated 5-HT protein incorporation was assessed culturing confluent 12 well tissue culture plates for the indicated period in the presence of 1 µCi [^3^H]-5-HT. After harvesting and three extensive washing steps with PBS, the cells were homogenized in ice-cold 300 mM PCA. Protein was collected by centrifugation and the supernatant was measured in a scintillation counter (Beckman) in 5 mL ReadyProtein scintillation cocktail (Beckman) to assess for the total [^3^H]-5-HT uptake. The protein pellets were washed four times with 300 mM PCA, boiled in 200 µL 10% SDS, and sample radioactivity was determined as above. Quantitative affinity precipitations were performed with cells grown in glucose-stimulation medium for 4 h in the presence of 1 µCi [^3^H]-5-HT. Lysates were obtained with Ni-NTA lysis buffer pH 8.0 (50 mM NaH_2_PO_4_, 300 mM NaCl, 10 mM imidazole, protease inhibitors w/o EDTA, 1% NP40, 1% Triton X-100, and 0.1% SDS). After separation of the debris by centrifugation, binding of 6xH-tagged Rabs was conducted with an excess of Ni-NTA magnetic beads (Qiagen; 20 µL beads/cells harvested from a confluent 12 well tissue culture cavity) and overhead turning at 4°C overnight. The beads were then washed three times (50 mM NaH_2_PO_4_ pH 8.0, 300 mM NaCl, 8 mM imidazole, protease inhibitors w/o EDTA, and 0.05% Tween 20) and used for quantification as above.

### Statistical Analyses

All data are presented as mean ± SEM and *p* values are from two-tailed Student's *t* tests type 3. Values of *p*<0.05 were considered as statistically significant.

## Supporting Information

Figure S1 ***Tph1*** **−/− mice are hypersensitive to the satiety mediating effect of systemically applied 5-HT.** (A) The central satiating effect of fenfluramine is normal. A dose of 1 mg kg^−1^ body weight was ineffective, whereas 3 mg kg^−1^ reduced food intake in a 2 h meal in both groups. (B) Peripheral effects of 5-HT differ between wt and *Tph1*−/− mice in a 1 h meal, pointing to a deregulated peripheral food intake regulation in the latter. *p<0.05; *n* = 11 to 13 per group. Data are presented as means ± SEM.
(0.52 MB EPS)

Figure S2 **Diabetes mellitus and β-cell dysfunction in** ***Tph1*** **−/− mice of two different genetic backgrounds.** (A–C) Glucose tolerance test of 35–38-wk-old *Tph1*−/− and wt mice (C57BL/6×129SvEvBrd intercross F11-generation). The shown data are from two independent experiments conducted with *n* = 9 and *n* = 6 mice per genotype. *p<0.05. (A) The fasting glucose levels are significantly elevated in these mice as compared to their wt littermates (9.1 versus 5.9 mM). *Tph1*−/− mice have also strongly elevated glucose levels after 30 min in glucose tolerance tests (2 g kg^−1^). (B) Fasting insulin levels are also significantly elevated but the insulin secretion in response to a glucose load was significantly shortened in *Tph1*−/− mice (best seen at the difference). (C) Glucose to insulin ratios are significantly increased after the glucose load, indicating a β-cell dysfunction. (D and E) A similar β-cell dysfunction and diabetes mellitus is seen in 12-wk-old *Tph1*−/− C57BL/6 backcross F8-generation mice. The experiment was conducted with *n* = 9 (wt) and *n* = 7 (*Tph1*−/−) mice. *p<0.05. Data are presented as means ± SEM.
(0.81 MB EPS)

Figure S3 **Biosynthesis and catabolism of 5-HT both offer possibilities to manipulate intracellular 5-HT contents.** (A) Application of the immediate 5-HT precursor 5-HTP bypasses the rate-limiting step of hydroxylation. In the cytoplasm, 5-HTP is rapidly converted to 5-HT by aromatic amino acid decarboxlase (AAAD). Monoamine oxidase (MAO) inhibitors impede the first step of the catabolism via 5-hydroxyindole acetaldehyde to 5-hydroxyindole acetic acid, leading to an accumulation of freshly synthesized 5-HT. (B) 5-HTP significantly elevates whole blood 5-HT levels in *Tph1*−/− mice. Subcutaneous injections with 5-HTP (50 mg kg^−1^) were performed twice a day for 3 d. The experiment was conducted with *n* = 8 (wt and *Tph1*−/− + 5-HTP) and *n* = 6 (*Tph1*−/−) mice. *p<0.005. Data are means ± SEM.
(0.61 MB EPS)

Figure S4 **5-HTP and pargyline largely increase intracellular 5-HT content.** RINm5F insulinoma cells were incubated in the presence of 500 µM 5-HTP or 20 µM pargyline for 4 h. After harvesting and three extensive washing steps, cells were homogenized in ice cold 300 mM PCA and analyzed for 5-HT metabolite content by reversed-phase HPLC. 5-HIAA, 5-hydroxyindole acetic acid. *p<0.05; *n* = 6. Data are presented as means ± SEM.
(0.57 MB EPS)

Figure S5 **VDCC stimulation, gap junctions, size, and HVA inward current component of** ***Tph1*** **−/− β-cells are normal in** ***Tph1*** **−/− pancreas slices, while LVA currents are increased.** (A) Peak amplitude of HVA Ca^2+^ currents (VACC) elicited by square pulse stimulation to −10 mV (*Tph1*+/+: *n* = 25; *Tph1*−/−: *n* = 18). Data are means ± SEM. (B) Whole-cell Ca^2+^ currents of *Tph1*+/+ (*n* = 24) and *Tph1*−/− (*n* = 19) β-cells evoked by voltage ramps ranging from −90 to +50 mV with duration of 300 ms (0.47 mV ms^−1^). The application of this protocol resulted in the separation of two inward current components, showing peaks around −30 and −5 mV, which most likely correspond to LVA and HVA Ca^2+^ currents. Data are presented as means. (C) Electrical coupling between the whole-cell patch-clamped β-cell and its immediate neighboring β-cell (*Tph1*+/+: *n* = 25; *Tph1*−/−: *n* = 18). Data are means ± SEM. (D) The resting membrane capacitance is a measure of the β-cell membrane surface area and does not differ significantly between the genotypes (*Tph1*+/+: *n* = 25; *Tph1*−/−: *n* = 18). Data are means ± SEM.
(0.65 MB EPS)

Figure S6 **5-HT restores slow component of Ca^2+^-dependent exocytosis of** ***Tph1*** **−/− β-cells.** (A) Amplitude of the fast component of the membrane capacitance change of single β-cells stimulated by ramp [Ca^2+^]i change. (B) Amplitude of the slow component of the membrane capacitance change. *p<0.05; **p<0.01. (C) Peak intracellular Ca^2+^ concentration during slow photo-release of NP-EGTA-caged Ca^2+^. Experiments were performed with *n* = 15 (*Tph1*−/−), *n* = 12 (*Tph1*−/− + 5-HTP_e_), *n* = 7 (*Tph1*−/− + 5-HT_i_, and *n* = 19 (wt). All data are presented as means ± SEM.
(0.69 MB EPS)

Figure S7 **Cysteamine inhibits protein serotonylation and reduces insulin secretion.** (A) Insulin secretion from RINm5F cells stimulated with 25 mM glucose in the presence of cysteamine (CTA; 500 µM; 3 h). *p<0.05; *n* = 3. Shown are combined data of three experiments conducted in quadruplicate. (B) Insulin secretion from INS-1 cells stimulated with 25 mM glucose in the presence of CTA (500 µM; 3 h). *p<0.05; *n* = 4. Data are means ± SEM.
(0.52 MB EPS)

Figure S8 **Quantitative Ni-NTA affinity precipitation of his-tagged Rabs from lysates of RINm5F cells stably transfected with corresponding constructs.** Immunoblotting demonstrates that the overexpressed proteins of the lysates (1/200th of input) are quantitatively precipitated, since AP supernatants (1/200th of input) lack the specific immuno-reactive bands, which are present in the lysates and largely enriched in the washed beads fractions (1/5th of total yield).
(1.06 MB TIF)

Figure S9 **The insulinoma cell lines β-TC3 and RINm5F express all components required for serotonylation in the insulin-secreting machinery.** Immunoblotting of cell lysates with commercially available antibodies. VMAT2, vesicular monoamine transporter 2.
(2.78 MB TIF)

Table S1 **Serological parameters of** ***Tph1*** **−/− mice.** The pancreas indicators α-amylase and lipase are normal. The elevated liver values are in line with our previous report about deficient liver regeneration capacity in *Tph1*−/− [11].
(0.04 MB DOC)

## Abbreviations

5-HT: serotonin
5-HTP: 5-hydroxytryptophan
HVA: high voltage activation
LVA: low voltage activation
MDC: monodansylcadaverine
MODY: maturity-onset diabetes of the young
SERT: 5-HT reuptake transporter
T1DM: type 1 diabetes mellitus
T2DM: type 2 diabetes mellitus
TGase: transglutaminase
*Tph1*−/−: tryptophan hydroxylase 1 knockout mice
wt: wild-type
VACC: voltage-activated Ca^2+^ channels
