# Supplementary figures and images for: Intracellular Serotonin Modulates Insulin Secretion from Pancreatic β-Cells by Protein Serotonylation
Source: PLoS Biol. 2009 Oct 27;7(10):e1000229. doi: 10.1371/journal.pbio.1000229 (PMC2760755; doi:10.1371/journal.pbio.1000229)

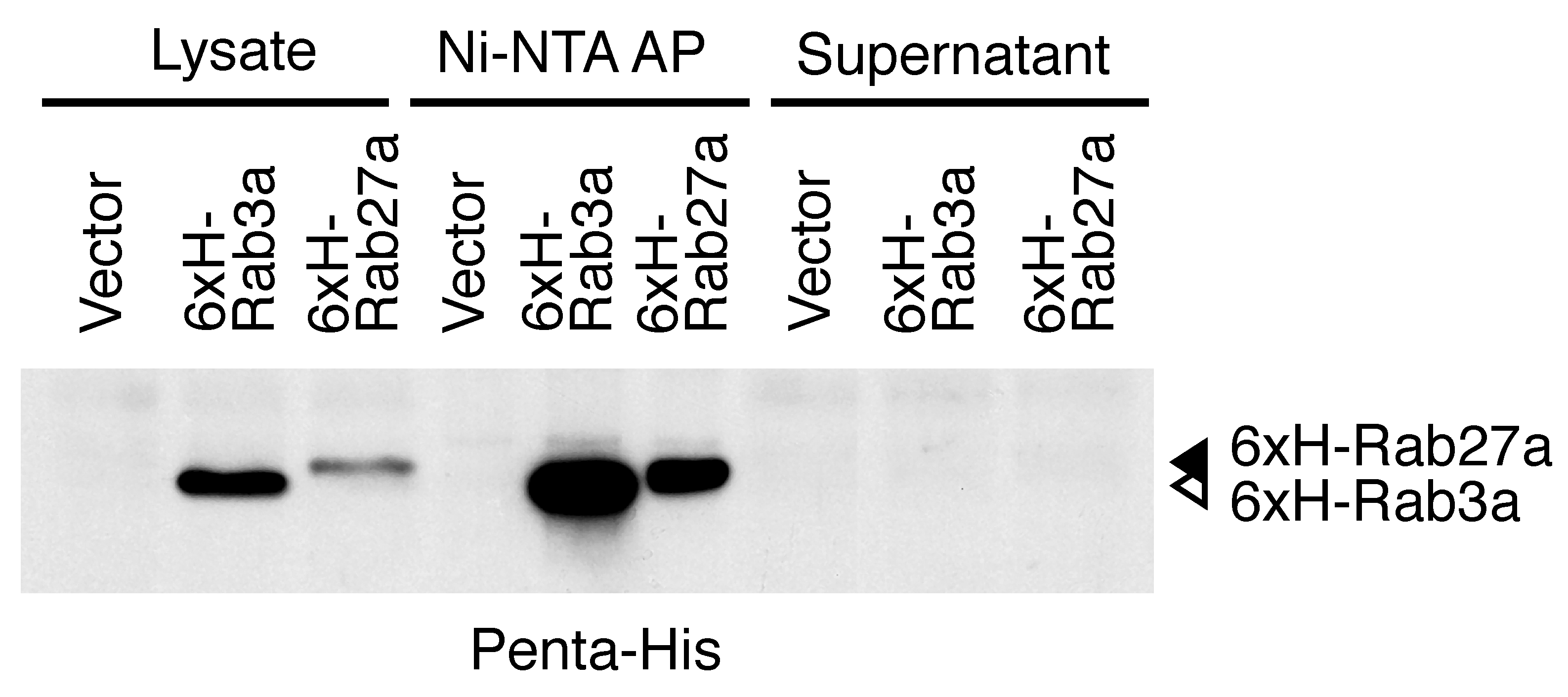

Supplement: Figure S8 — Quantitative Ni-NTA affinity precipitation of his-tagged Rabs from lysates of RINm5F cells stably transfected with corresponding constructs. Immunoblotting demonstrates that the overexpressed proteins of the lysates (1/200th of input) are quantitatively precipitated, since AP supernatants (1/200th of input) lack the specific immuno-reactive bands, which are present in the lysates and largely enriched in the washed beads fractions (1/5th of total yield). (1.06 MB TIF) [file pbio.1000229.s008.tif]

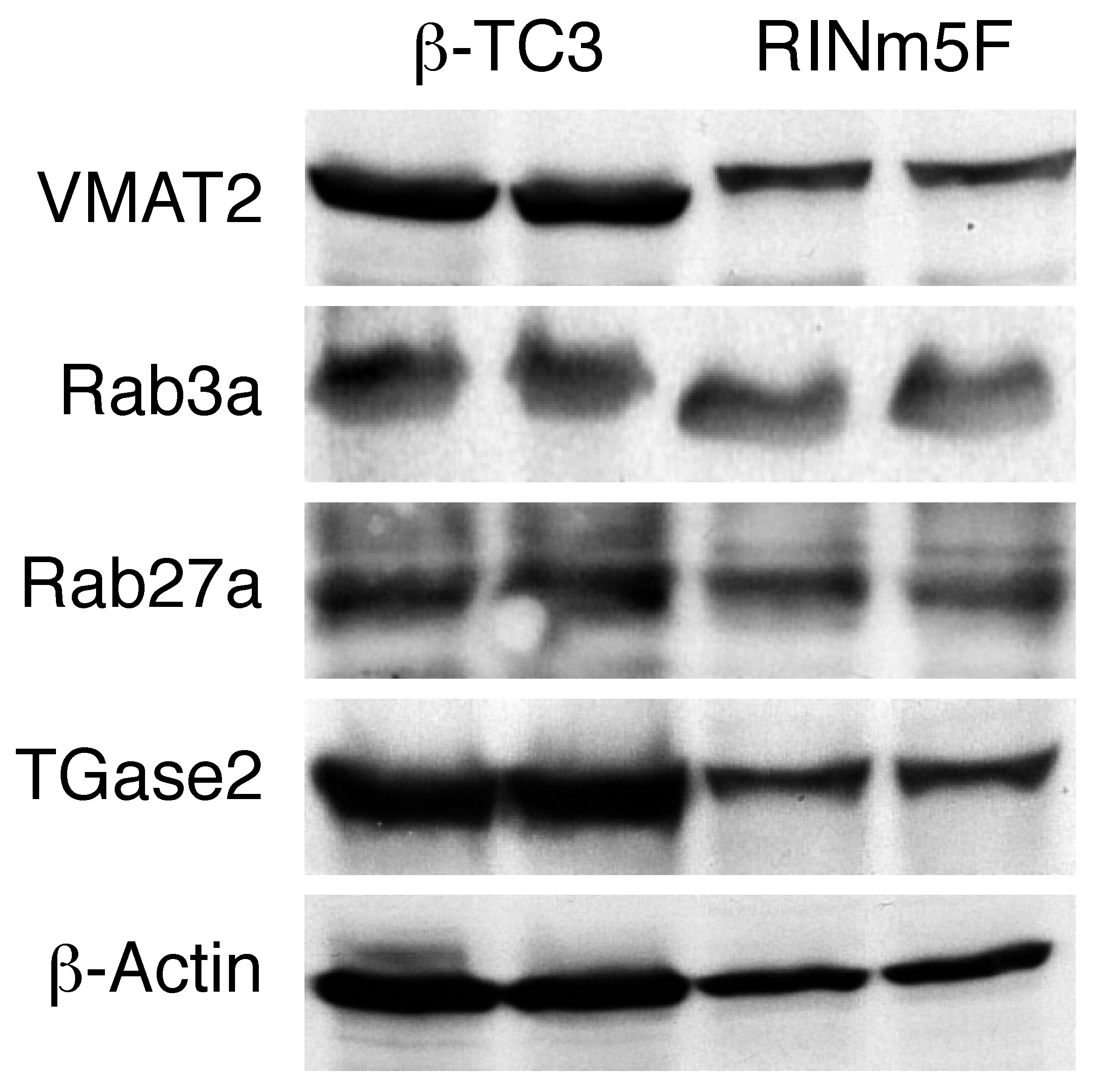

Supplement: Figure S9 — The insulinoma cell lines β-TC3 and RINm5F express all components required for serotonylation in the insulin-secreting machinery. Immunoblotting of cell lysates with commercially available antibodies. VMAT2, vesicular monoamine transporter 2. (2.78 MB TIF) [file pbio.1000229.s009.tif]
