# Supplementary material for: Intracellular Serotonin Modulates Insulin Secretion from Pancreatic β-Cells by Protein Serotonylation
Source: PLoS Biol. 2009 Oct 27;7(10):e1000229. doi: 10.1371/journal.pbio.1000229 (PMC2760755; doi:10.1371/journal.pbio.1000229)
Supplement: Table S1 — Serological parameters of Tph1 −/− mice. The pancreas indicators α-amylase and lipase are normal. The elevated liver values are in line with our previous report about deficient liver regeneration capacity in Tph1−/− [11]. (0.04 MB DOC) [file pbio.1000229.s010.doc]

| Clinical parameter | *Tph1*+/+^(a)^ | *Tph1*-/-^(a)^ | Units | t-test2;3 |
| --- | --- | --- | --- | --- |
| Urea | 27 ± 3 | 26 ± 7 | mg/dL | N.S. |
| Total serum protein^c^ | 3.8 ± 0.2 | 4.1 ± 0.2 | g/dL | p < 0.007 |
| Serum albumin^c^ | 2.4 ± 0.1 | 2.6 ± 0.1 | g/dL | p < 0.005 |
| Total bilirubin | 0.13 ± 0.01 | 0.14 ± 0.02 | mg/dL | N.S. |
| ALT (GPT)^c^ | 18 ± 2 | 31 ± 9 | U/L | p < 0.001 |
| AST (GOT)^c^ | 44 ± 3 | 59 ± 9 | U/L | p < 0.001 |
| Alk. phosphatase | 102 ± 33 | 108 ± 27 | U/L | N.S. |
| GLDH^c^ | 5 ± 1 | 8 ± 3 | U/L | p < 0.03 |
| Bile acids | 2.8 ± 0.7 | 2.4 ± 0.7 | µmol/L | N.S. |
| α-amylase^b^ | 2225 ± 176 | 2232 ± 381 | U/L | N.S. |
| Lipase^b^ | 19 ± 3 | 20 ± 6 | U/L | N.S. |
| Cholesterol^d^ | 60 ± 5 | 58 ± 9 | mg/dL | N.S. |
| Total triglycerides^d^ | 69 ± 11 | 76 ± 16 | mg/dL | N.S. |
|  |  |  |  |  |

^a^n = 15 mice; ^b^pancreas indicators; ^c^elevated liver values; ^d^normal lipid values; N.S.: not significant.
